# Population-Level Impact of Active Tuberculosis Case Finding in an Asian Megacity

**DOI:** 10.1371/journal.pone.0077517

**Published:** 2013-10-16

**Authors:** David W. Dowdy, Ismat Lotia, Andrew S. Azman, Jacob Creswell, Suvanand Sahu, Aamir J. Khan

**Affiliations:** 1 Department of Epidemiology, Johns Hopkins Bloomberg School of Public Health, Baltimore, Maryland, United States of America; 2 Center for Tuberculosis Research, Johns Hopkins University, Baltimore, Maryland, United States of America; 3 Interactive Research and Development, Karachi, Sindh, Pakistan; 4 Stop TB Partnership, Geneva, Switzerland; 5 Department of International Health, Johns Hopkins Bloomberg School of Public Health, Baltimore, Maryland, United States of America; London School of Hygiene and Tropical Medicine, United Kingdom

## Abstract

**Background:**

The potential population-level impact of private-sector initiatives for tuberculosis (TB) case finding in Southeast Asia remains uncertain. In 2011, the Indus Hospital TB Control Program in Karachi, Pakistan, undertook an aggressive case-finding campaign that doubled notification rates, providing an opportunity to investigate potential population-level effects.

**Methods:**

We constructed an age-structured compartmental model of TB in the intervention area. We fit the model using field and literature data, assuming that TB incidence equaled the estimated nationwide incidence in Pakistan (primary analysis), or 1.5 times greater (high-incidence scenario). We modeled the intervention as an increase in the rate of formal-sector TB diagnosis and evaluated the potential impact of sustaining this rate for five years.

**Results:**

In the primary analysis, the five-year intervention averted 24% (95% uncertainty range, UR: 18-30%) of five-year cumulative TB cases and 52% (95% UR: 45-57%) of cumulative TB deaths. Corresponding reductions in the high-incidence scenario were 12% (95% UR: 8-17%) and 27% (95% UR: 21-34%), although the absolute number of lives saved was higher. At the end of five years, TB notification rates in the primary analysis were below their 2010 baseline, incidence had dropped by 45%, and annual mortality had fallen by 72%. About half of the cumulative impact on incidence and mortality could be achieved with a one-year intervention.

**Conclusions:**

Sustained, multifaceted, and innovative approaches to TB case-finding in Asian megacities can have substantial community-wide epidemiological impact.

## Introduction

An estimated 12 million people worldwide have active tuberculosis (TB); less than half of these individuals will be notified to public health authorities within a year [1]. Case finding strategies that rely on passive presentation are therefore unlikely to achieve ambitious goals for TB elimination [2,3]. Many interventions have sought to improve TB case finding [4-6], but assessing their population-level impact on TB epidemiology remains difficult [7]. A recent randomized trial of community-based active TB case finding in Zambia and South Africa found a marginally significant 22% reduction in TB prevalence, but the parallel “enhanced case finding” arm of that trial (arguably a more intensive intervention) found no effect [8]. A separate trial in Zimbabwe described a 40% reduction in prevalence over three years [9], but this reduction was not in reference to a control group, so secular time trends could not be excluded. However, both of these studies required resource-intensive efforts (e.g., door-to-door sputum collection) that are difficult to implement on a population scale. Furthermore, the only large trials of wide-scale active TB case-finding come from sub-Saharan Africa; these results may not generalize to Southeast Asia (which accounts for nearly half of the world’s TB deaths [1]), where many people seek care in the poorly-regulated private sector [10-12].

A recent study evaluated the impact of an aggressive approach to active TB case finding centered at the Indus Hospital TB Control Program in Karachi, Pakistan [13]. In this program, a communications campaign was combined with mobile phone-based conditional cash transfers to screeners at local private sector clinics; over the year in which the program was implemented, TB case notifications doubled. This intervention may serve as a model for similar combined case-finding interventions in other megacities, but decisions to scale up such interventions require estimates of their anticipated impact on TB incidence and mortality in the population. To help inform these decisions, we constructed a mathematical model of the TB epidemic in the area covered by this intervention.

## Methods

### Study Description

The study intervention has been described in detail elsewhere [13]. Briefly, in 2011, a year-long mass communications initiative was deployed in a section of Karachi, Pakistan (the intervention area), encouraging people with prolonged cough (>2 weeks) to seek care at local family clinics or Indus Hospital – a 150-bed, private hospital that provides free care and notified 41% of all TB cases in the intervention area in 2010. The initiative included billboards, cable television ads, posters, and flyers, as well as deployment of local residents as TB screeners in over 50 local private sector family clinics. Screeners were provided a mobile phone, then given a monthly stipend and small conditional cash transfers via phone bank transfer for such activities as submitting a daily report, procuring an acceptable sputum sample, and identifying a smear-positive case. Between 2010 and 2011, the number of notified TB cases (i.e., individuals starting treatment) in the intervention area rose from 1569 to 3140, while the corresponding number in a nearby control area remained stable (876 notifications in 2010, 818 in 2011). Of all notifications at Indus Hospital in 2011, 9% were listed as “transfer out,” suggesting that most patients were retained in the Indus Hospital TB Program for the duration of their treatment. We employed detailed data from this study to populate an epidemic model of TB.

### Model Structure

**Figure 1:**
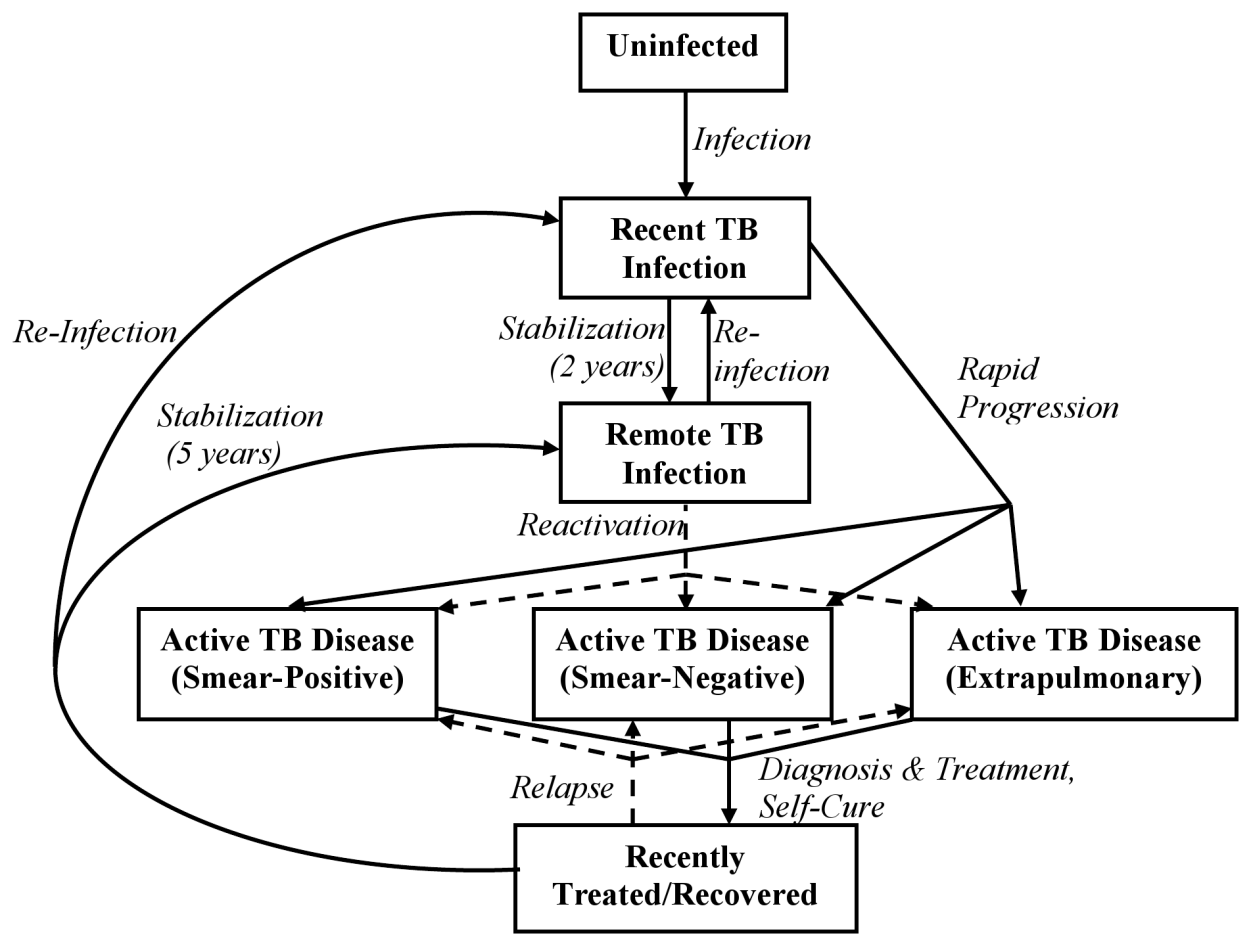
Transmission Model of Tuberculosis in Karachi, Pakistan. Boxes represent compartments in the model, and arrows represent rates of flow, which are modeled as ordinary differential equations. Not shown here, but included in the model, are 9 age classes and TB-specific mortality occurring from each active TB compartment.

Using ordinary differential equations, we developed a compartmental model of the TB epidemic in the intervention area. Our primary aim was to project the five-year impact of the study intervention on TB incidence, undiagnosed prevalence, mortality, and case detection. We utilized a parsimonious modeling approach in order to minimize assumptions (Figure 1). The model describes sub-populations as having no TB infection, latent TB infection (recently infected or remotely infected), active TB disease (smear-positive pulmonary, smear-negative pulmonary, or extrapulmonary), and recently treated/recovered TB disease. Individuals with smear-negative pulmonary TB are considered infectious (at a reduced level), whereas individuals with latent TB, treated/recovered TB, and extrapulmonary TB are presumed non-infectious. We modeled treatment as having immediate effectiveness, transitioning people into a “recently treated” compartment with increased risk of relapse [14,15]. We assumed that people remain at elevated risk of active TB for at least two years following infection [16].

We modeled age in nine strata (one stratum for children ages 0-9, and every five years thereafter until the age of 50) to allow for an age structure similar to that seen in Karachi. We found no substantive changes when using a model with two age classes (children and adults). In our study site, 57% of pediatric TB occurred in children ages 10-14; these adolescents presented most commonly with pulmonary TB and were modeled as adults. By contrast, the focus of this intervention was on chronic cough, and most TB in children age 0-9 was non-pulmonary, so we did not enumerate these cases (6% of all cases) in the model, nor did we consider them infectious. We assumed population growth of 3% per year [17] and did not incorporate human immunodeficiency virus (HIV) infection, as less than 1% of TB in Pakistan is HIV-related [1]. Parameter values appear in Table 1, and further details are given in the Supporting Information S1.

**Table 1:** Parameters for Model of TB Case Finding in Karachi, Pakistan.

**Model Parameter**	**Value in Model**	**Sensitivity Analysis Range**	**References**
Number of secondary infections per smear-positive person-year	16.1^a^	14-18	Fit to incidence in Pakistan[1]
Relative infectiousness of smear-negative cases	0.22	0.16-0.32	[22]
Relative rate of progression to high-risk (early) infection, latent TB	0.50	0.2-0.8	[16,23,24]
Rate of rapid progression during first two years after infection, per year	0.07	0.05-0.09	[16]
Rate of slow progression of remote TB infection, per year	0.0005	0.0003-0.0007	[25]
Proportion of incident TB that is extrapulmonary	0.24	0.1-0.3	Study data
Proportion of incident pulmonary TB that is smear-positive	0.65	0.55-0.7	Study data, [26,27]
Mortality rate per year, smear-positive TB	0.23	0.15-0.3	[28]
Mortality rate per year, smear-negative and extrapulmonary TB	0.07	0.05-0.15	[28]
Natural cure rate per year, smear-positive TB	0.10	0.05-0.15	[28]
Natural cure rate per year, smear-negative and extrapulmonary TB	0.27	0.15-0.4	[28]
Size of informal sector that does not notify TB, relative to notifications	0.455	0-1.0	[19]
Treatment success proportion in 2008, notified cases	0.75	0.65-0.85	Study data
Treatment success proportion, informal sector	0.5	0-0.7	[29]
Relapse rate, per year	0.024^a^	0-0.05	Study data^c^

^a^ In an alternative “high-incidence” scenario, fit to an incidence of 350 per 100,000 but with the same notification rates, the number of infections per smear-positive person-year was 13.8, and the relapse rate was 0.029 per year.

^b^ Assumes that proportional reductions in treatments in the informal sector mirrored reductions in notifications from formal-sector clinics in the intervention area that were not affiliated with Indus Hospital (the focus of the intervention).

^c^ Fit to the proportion of incident cases arising from previously treated individuals.

### Baseline Scenarios

We fit the model to two different baseline scenarios, a primary analysis with an underlying TB incidence rate representative of the World Health Organizations (WHO)-estimated TB incidence in Pakistan (231 per 100,000/year) [1], and a high-incidence scenario with an underlying TB incidence that was 1·5 times higher (350 per 100,000/year). The primary analysis is consistent with the similar per-capita TB incidence in urban and rural areas found in Vietnam [18].

A sizeable proportion of TB in Pakistan is diagnosed and treated by providers who do not notify cases to public authorities. We labeled these providers the “informal sector,” distinguished from the “private sector” because some private providers (e.g., at Indus Hospital) notify cases routinely. To estimate the size of the informal sector, we took the total estimated size of the private market in Pakistan (45% of all treatments) [19], and subtracted the estimated number of notifications made by private providers (20% of all notifications) [20].

### Diagnostic Rate

We modeled TB diagnosis as a constant rate (hazard) of case detection applied throughout the infectious period. This “diagnostic rate” is a theoretical construct that includes all efforts at diagnosis, defined as (1/mean duration of infectiousness before diagnosis) among all TB cases in the community who are effectively diagnosed. The diagnostic rate differs from the classically-defined “case detection rate,” defined as the proportion of all people with active TB who are notified. We multiplied the diagnostic rate by the treatment success proportion to estimate the rate of successful treatment of active TB, thereby assuming that unsuccessfully treated cases remain infectious. We modeled separate diagnostic rates for the different clinical manifestations of TB (smear-positive pulmonary, smear-negative pulmonary, extrapulmonary), fitting each rate to the number of notified TB cases in the study region after accounting for the probability of successful diagnosis and treatment in the informal sector. 

### Model Calibration and Intervention Scenarios

We assumed a steady-state population in 2008 and fit each model to its estimated TB incidence and observed number of TB notifications in that year. We brought each model to equilibrium using an iterative process whereby we simultaneously varied the TB transmission rate, relapse rate, and TB diagnosis rates (by clinical manifestation) until the model reproduced the target TB incidence rate, observed proportion of retreatment cases, and observed case notification rates (by clinical manifestation). In 2009 and 2010, prior to the start of the intervention, two secular trends were visible: (a) increase in case notifications at Indus Hospital (with no significant change in notifications at other sites, likely due to the growing reputation of Indus Hospital, a new facility that opened in 2007); and (b) decline in TB treatment success proportion at Indus Hospital. Preliminary data from Indus Hospital suggest that treatment success during the intervention (2011-2012) returned to the higher levels seen in 2008, but since it is unlikely that treatment success will improve in other settings with a dramatic rise in patient volume, we conservatively modeled treatment success as stable at 2010 levels throughout the intervention. We therefore fit the diagnostic rate and treatment success proportion to linear changes over this time period. 

We conceptualized the intervention as a comprehensive case-finding package, rather than specifying any one activity. We modeled the effect of this package as a linear increase in each type-specific TB diagnostic rate (smear-positive, smear-negative, and extrapulmonary) over the course of 2011, fit to the observed number of notified type-specific cases in 2011. Due to the seasonality of notification data in Pakistan, we did not model month-over-month rates. On January 1, 2012, we stabilized the diagnostic rate, thereby assuming that ongoing active case-finding activities could maintain the mean duration of active TB (prior to diagnosis) in the intervention area at this level. Preliminary data from 2012 suggest that the case notification rate in the intervention is remaining stable. In sensitivity analysis, we assumed a linear decline in the diagnostic rate from a peak in January 1, 2012, to return to the rate in the counterfactual scenario on January 1, 2013 (i.e., a one-year increase in case-finding activities, followed by a one-year decline back to baseline, with no persistent effect on diagnostic rate beyond that time), as well as intermediate durations of effect between one and five years. Our primary outcomes were the cumulative TB incidence and mortality in the intervention area over the five-year period from 2011 through 2015, comparing the intervention scenario (observed) against the counterfactual scenario in which no intervention was deployed. We also estimated the prevalence of undiagnosed TB and the number of TB cases detected (i.e., burden on the health system) under both intervention and counterfactual scenarios. 

We modeled the counterfactual scenario as having a TB diagnostic rate that increased over 2011, but at a progressively slower rate such that, by January 1, 2012, the diagnostic rate stabilized, thus allowing a fair comparison between the intervention and counterfactual scenarios. Since the intervention focused on case detection, we assumed that the treatment success proportion remained stable beyond 2010 in both scenarios. 

### Informal Sector Diagnoses

A number of the “new” notifications observed after implementation of the intervention represent individuals who, in the counterfactual scenario, are diagnosed and treated in the informal sector. Since the number of such “crossovers” is unknown, we adopted a maximally conservative strategy in which the size of the informal TB sector declined linearly to zero over 2011 in the intervention scenario. Thus, by January 1, 2012, we assumed that notifications accounted for all TB cases treated in the intervention scenario, while assuming that informal-sector treatments remain constant in the counterfactual scenario. To the extent that informal-sector treatments occur in the intervention scenario, our model underestimates the total number of cases treated and thereby underestimates the population-level impact of the intervention.

### Sensitivity and Uncertainty Analyses

We performed both one-way sensitivity analyses and probabilistic uncertainty analyses, including all model parameters in Table 1. For the latter, we varied all variables simultaneously using Latin Hypercube sampling across beta distributions defined by the upper and lower bounds in Table 1, with the alpha parameter set to 4.0 and the most likely value defining the mode. This analysis was used to estimate 95% uncertainty ranges (95% UR), defined by the 2.5^th^ and 97.5^th^ percentile of results carried out over 1,000 simulations. The model was independently constructed in parallel by two authors (DWD and ASA), with differences in model specification resolved by consensus until results converged. We solved differential equations using the deSolve package in R, version 2.13.1 (R Foundation for Statistical Computing, 2011) with outcomes computed using a time step of 0.1 year. Open-source model code is available on a public website (https://github.com/ddowdy/KarachiACFmodel).

## Results

### Pre-intervention

In 2008, the intervention area had a population of 838,057, and 916 cases of TB were notified (notification rate 109 per 100,000/year), of whom 491 (54%) were smear-positive. At steady-state, given the parameter assumptions in Table 1, this notification rate corresponds to an undiagnosed TB prevalence of 327 per 100,000 in the primary analysis (underlying TB incidence = 231 per 100,000/year) and 660 per 100,000 in the high-incidence scenario (incidence = 350 per 100,000/year). The estimated annual risk of TB infection among uninfected individuals was 2.8% in the primary analysis and 4.9% in the high-incidence scenario. The corresponding TB diagnostic rates (and proportion of TB cases notified, i.e., “case detection rate”) in the primary analysis were 0.41/year (44%) for smear-positive pulmonary TB, 0.16/year (28%) for smear-negative pulmonary TB, and 0.50/year (47%) for extrapulmonary TB. An additional estimated 31% of TB diagnoses were made in the informal sector (i.e., not notified), and diagnostic rates were 43-56% lower in the high-incidence scenario. By 2010, the TB notification rate had increased to 176 per 100,000/year in the primary analysis, and the smear-positive diagnostic rate had increased to 0.85/year, with 62% of smear-positive cases notified. 

### Post-Intervention

**Figure 2:**
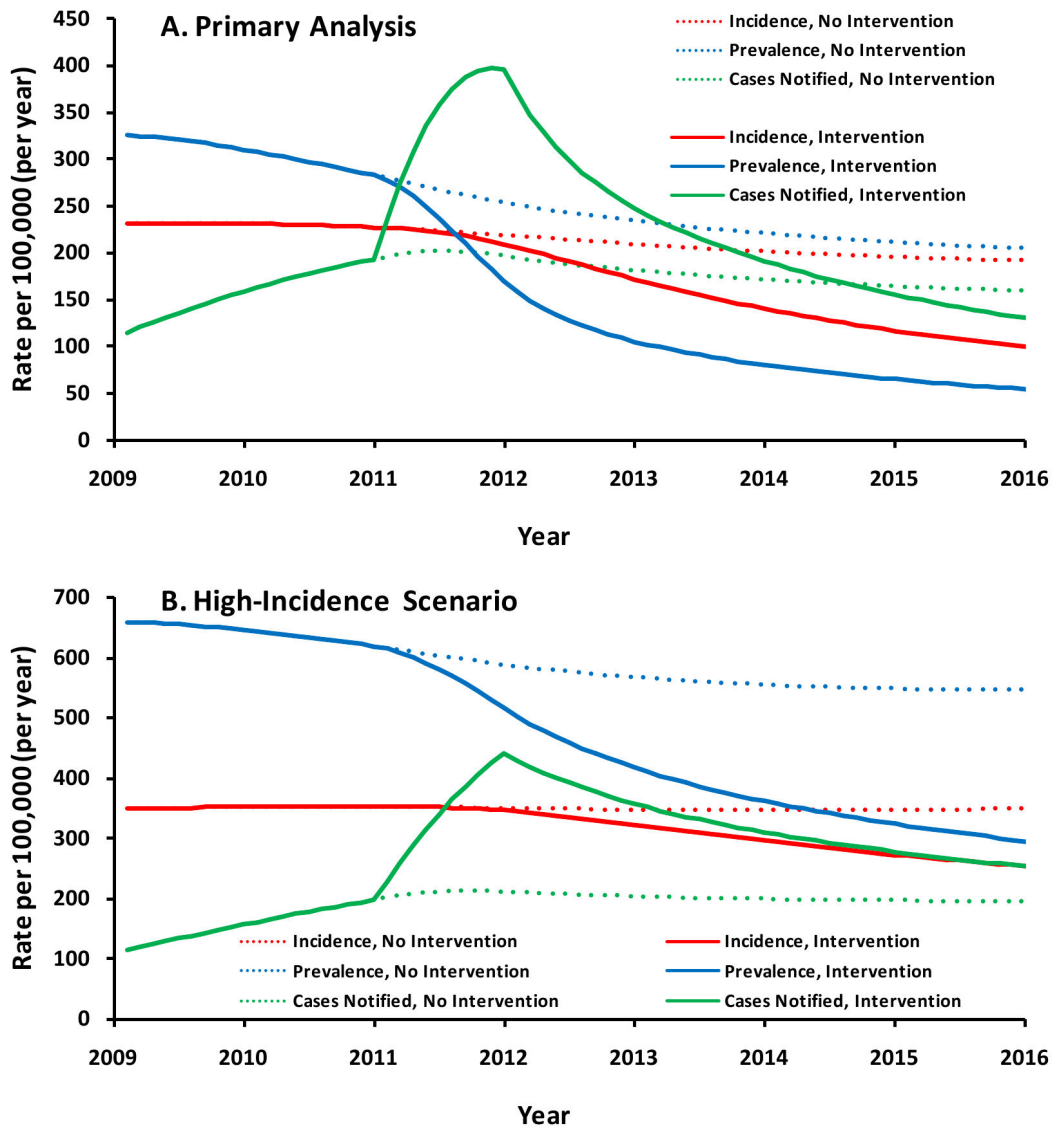
Projected TB Epidemics After Implementation of a Case-Finding Intervention in Karachi, Pakistan. TB incidence is shown in red, prevalence in blue, and notification rates in green. Dotted lines represent the counterfactual scenario of no intervention, whereas solid lines presume that the intervention is maintained at equal intensity for five years. The primary analysis assumes an underlying TB incidence of 231 per 100,000/year (representative of Pakistan [1]) in 2008, whereas the high-incidence scenario assumes a baseline incidence of 350 per 100,000/year. In the primary analysis, case notifications rise transiently above both incidence and prevalence because both incident and prevalent cases are simultaneously being detected and treated.

In 2011, after implementation of the intervention, the TB notification rate increased to 343 per 100,000/year, corresponding to an absolute increase in TB detection of 1.94/year (new case detection proportion, 89%) for smear-positive pulmonary TB; thus, the new diagnostic rate for smear-positive TB was 0.85 + 1.94 = 2.79/year. Corresponding increases in the diagnostic rate from 2010 to 2011 (and new case detection proportions by the end of 2011) were 1.26/year (75%) for smear-negative pulmonary TB and 2.04/year (87%) for extrapulmonary TB in the primary analysis (see also Table S3 in Supporting Information S1). The modeled 95% uncertainty range (UR) for TB case notification in the primary analysis was 267-430 per 100,000/year, corresponding closely to the observed notification rates in the quarters of 2011 with the lowest and highest numbers of TB notifications (269 and 431, respectively). In the absence of the intervention (counterfactual scenario), the projected total number of incident TB cases from 2011 through 2015 was 9,900 (95% UR: 6,900-14,900) in the primary analysis and 16,800 (95%UR: 11,500-25,600) in the high-incidence scenario; corresponding numbers of TB deaths were 1,500 (95%UR: 1,100-2,400) and 3,900 (95%UR: 2,900-5,900). The intervention averted an estimated 24% of cases (95% UR: 18-30%) and 52% of deaths (95% UR: 45-57%) in the primary analysis and 12% of cases (95% UR: 8-17%) and 27% of deaths (95% UR: 21-34%) in the high-incidence scenario (Table 2). Although the proportional reduction in mortality was lower in the high-incidence scenario than in the primary analysis, the absolute number of estimated lives saved was greater. Both incident and prevalent cases were notified and treated during the intervention, such that the case notification rate rose transiently above either incidence or prevalence alone (Figure 2). If the intervention could be sustained at equal intensity for five years in the primary analysis, we projected that notification rates would fall below the no-intervention baseline (by removing prevalent cases) in 2014 (Figure 2), and that by 2015, TB incidence would be reduced by 45% (95% UR: 36-53%), and both prevalence and mortality by 72% (95% UR: 65-77%), relative to the no-intervention baseline (Table 2).

**Table 2:** Projected Cumulative Epidemiological Impact of a TB Case-Finding Intervention in Karachi, Pakistan: 2011-2015^**a**^.

**Scenario**	**TB Incidence**		**TB Mortality**	
	*Cases*	*Reduction^b^*	*Deaths*	*Reduction^b^*
Primary Analysis (TB incidence 231 per 100,000/year in 2008)				
No Intervention				
Year 5 (2015)	2,000		290	
Cumulative (2011-2015)	9,900		1,500	
Intervention				
Year 5 (2015)	1,100	900 (45%)	80	210 (72%)
		95% UR: 36-53%		95% UR: 65-77%
Cumulative (2011-2015)	7,600	2,300 (24%)	740	790 (52%)
		95% UR: 18-30%		95% UR: 45-57%
High-Incidence Scenario (TB incidence 350 per 100,000/year in 2008)				
No Intervention				
Year 5 (2015)	3,600		800	
Cumulative (2011-2015)	16,800		3,900	
Intervention				
Year 5 (2015)	2,700	890 (25%)	460	350 (43%)
		95% UR: 18-32%		95% UR: 33-51%
Cumulative (2011-2015)	14,700	2,000 (12%)	2,900	1,100 (27%)
		95% UR: 8-17%		95% UR: 21-34%

UR, uncertainty range

^a^ Total population 838,057 in 2008; population growth 3% per year

^b^ Relative to the corresponding no-intervention (counterfactual) scenario

### Sensitivity Analysis

**Figure 3:**
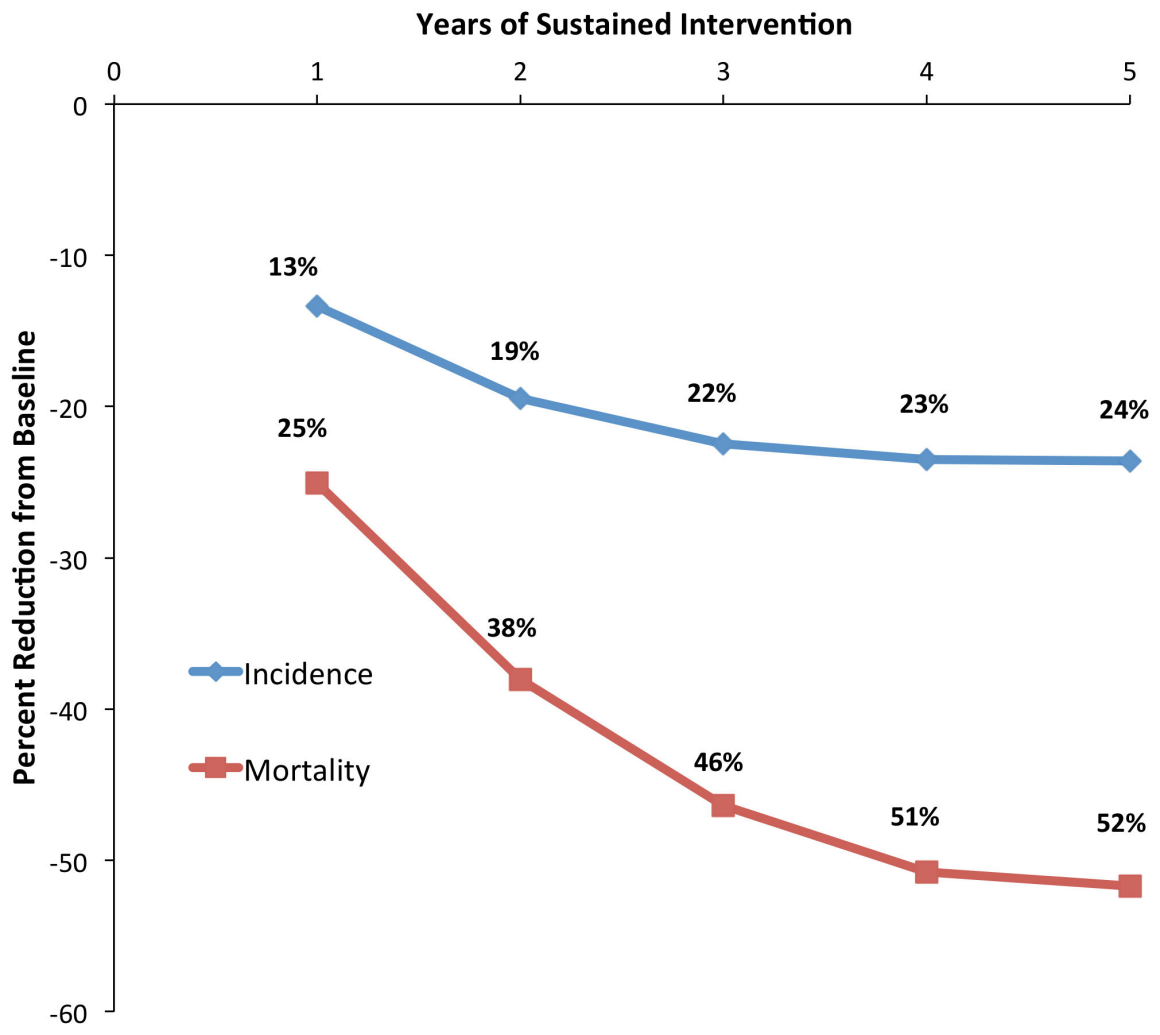
Relationship Between Duration of Intervention and Impact on Cumulative Five-Year Outcomes. The x-axis shows the duration of the intervention, scaled up to a maximum TB diagnostic rate over one year, assuming that the diagnostic rate then declines back to baseline over the subsequent year. Thus, a one-year duration corresponds to a single time step at maximum diagnostic rate, preceded by one year of scale-up and followed by one year of gradual return to baseline, then three years of baseline diagnostic intensity. The y-axis shows cumulative percent reduction in five-year incidence and mortality. The five-year intervention corresponds to the primary analysis reported in the manuscript text.

We first considered the scenario in which the intervention impact lasted only one year, with the diagnostic rate falling back to baseline by January 1, 2013. In this scenario, the projected reduction in cumulative incidence (2011-2015) was cut approximately in half (from 24% to 13% in the primary analysis and 12% to 6% in the high-incidence scenario), as was the reduction in mortality (52% to 25%, 27% to 11%). Figure 3 shows the relationship between sustained duration of the intervention and projected reduction in incidence and mortality. We also considered a scenario in which the entire increase in smear-unknown, smear-negative, and extrapulmonary TB notification in 2011 and beyond was due to false-positive diagnosis. In this scenario, the projected intervention impact, measured as the reduction in cumulative incidence and mortality in the primary analysis, was attenuated from 24% to 11% (incidence) and from 52% to 22% (mortality). 

**Figure 4:**
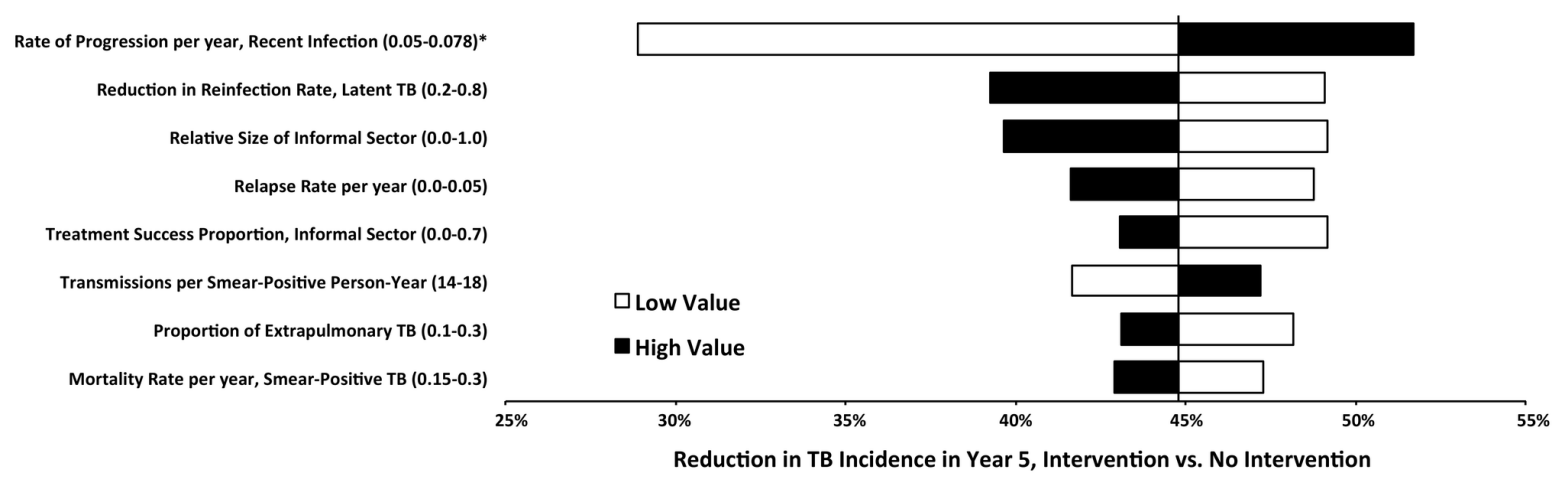
One-Way Sensitivity Analysis: Reduction in TB Incidence during 2015, Primary Analysis. In the primary analysis, the intervention was projected to reduce TB incidence by 45% relative to no intervention in 2015 (vertical line, also seen in Table 2). White bars show the projected reduction assuming the low value of each parameter, and black bars show the corresponding reduction assuming the high value. Only those parameters for which the projected impact on incidence varied by ±2% are shown here. * Changes in the rate of rapid progression were balanced by corresponding changes in the rate of slow progression (reactivation) to maintain constant incidence; the low value corresponds to 61% of active TB due to recent infection, versus 85% at baseline and 100% at the high value.

In one-way sensitivity analysis among model parameters in Table 1, the projected impact of the intervention on TB incidence in 2015 was most sensitive to parameters that defined the number of new TB cases expected to occur as a result of recent transmission (Figure 4). Reducing the proportion of TB due to recent infection at baseline (by varying the balance between fast and slow progression while maintaining stable incidence) from 85% to 61% attenuated the reduction in TB incidence during 2015 from 45% to 29% in the primary analysis (Figure 3, upper white bar). No other one-way sensitivity analysis changed the projected reduction in incidence by more than ±6.0% (i.e., outside the range of 39-50%). The impact of the intervention was only moderately sensitive to assumptions about the extent of informal-sector TB treatment; when we assumed an informal sector that equaled the size of the formal sector and was completely subsumed upon deployment of the intervention, the modeled intervention still reduced TB incidence by 40% in 2015. Sensitivity analyses of cumulative 5-year incidence and mortality – including both the primary analysis and high-incidence scenario – were qualitatively similar, with impact on mortality being somewhat more robust to parameter variation (data not shown).

## Discussion

Using data from a corresponding field evaluation, this epidemic model projects that aggressive, innovative, and multifaceted approaches to active case finding could have dramatic population-level impact on local TB epidemics. If its effects on diagnostic rates were sustained over a five-year time frame, this private-sector, communications-oriented intervention could avert 2300 TB cases and save 790 lives in an area with an average population of about one million; if the effects lasted only one year, half of this impact could still be achieved. By the end of five years, the sustained intervention could reduce TB incidence by 45%, TB mortality by 72%, and number of patients treated for TB in the formal sector below the counterfactual scenario of no intervention. The intervention had less relative impact, but saved more lives, in an alternative analysis in which the underlying incidence was higher. Intensive, multifaceted, and sustained efforts to improve TB case finding may rapidly and dramatically reduce TB burden in Southeast Asian megacities.

Our model assumes a high rate of ongoing infection as may be true in densely populated urban areas. We estimated an annual risk of infection in the primary analysis similar to the upper range of that measured in Zambia in 2005 [30] (estimated TB prevalence 411 per 100,000 [1]) and to historical estimates in the Southeast Asia region [31]. Our projections of impact are similar to those of Corbett and colleagues [9], who demonstrated a 40% reduction in TB prevalence after six rounds of mobile van-based active case-finding over three years in Harare, Zimbabwe – although these before-after results are likely not entirely attributable to the intervention. We projected a 40% cumulative reduction in TB prevalence achievable at the same point in time, suggesting that similar epidemiological impact can be attained from active case finding efforts that are targeted to diverse epidemiological situations, provided that the proportion of active TB due to recent infection (rather than reactivation) is high. The ZAMSTAR study [21] found a smaller epidemiological impact (22% reduction in prevalence) from a more intensive intervention of household counseling over three years; its “enhanced” case-finding arm (which more closely resembles the intervention in this study, though through the public rather than private sector) showed no effect on TB prevalence. Both of these studies were performed in areas with far less private-sector involvement in TB control; corresponding large randomized trials of TB case-finding from Southeast Asia are lacking, making models such as this one critical for providing preliminary estimates of impact. 

Compared to other modeling studies of active TB case finding, our results suggest similar to somewhat greater effect. For example, Murray and Salomon [32] projected that screening of a population with mass miniature radiography every seven years could avert 22% of TB cases, and Dodd and colleagues estimated that periodic active case finding could reduce TB incidence by one-third or more [33]. We emphasize, however, that results from a communications-based campaign in an Asian megacity may not directly compare to those from models of more generic interventions in context-free populations.

The intervention studied here focused primarily on engaging the private sector and achieved a dramatic effect even with worst-case assumptions about the extent to which a communications intervention might simply shift the burden of TB diagnosis and treatment from the informal to the formal sector. Varying the size of the informal sector to 100% of the formal sector did not reduce the projected impact of the intervention on TB incidence by more than 10% (Figure 3), partially because treatment outcomes were assumed to be better in the formal sector. Ultimately, while the size of the informal sector in Karachi is unknown, assumptions about the informal sector do not drive the model’s conclusions.

Our sensitivity analysis suggests that case-finding interventions may have greatest population-level impact in areas where recent transmission drives TB incidence. Thus, densely-populated megacities may represent an ideal setting for their deployment. Not only are transmission rates high and existing diagnostic rates low in these settings, but low-cost mobile phones and local media and communications campaigns can reach large numbers of patients and private providers with relatively limited geographic span. Such campaigns are also likely to prove cost-effective, if not cost-saving, in the long term as the number of individuals requiring TB treatment may decline below baseline levels. In the short term, however, a massive influx of individuals requiring treatment may overwhelm TB programs that are already facing severe shortages of resources, with possible negative impacts on follow-up, treatment completion, and passive case detection. Preliminary data from Indus Hospital suggest that treatment success rates did not decline during the period of the intervention. However, given the delicacy of this balance between shorter-term capacity and the potential for longer-term gains, our results support the need for randomized implementation trials of similar interventions in other Asian megacities, with long-term follow-up of population-level impact (e.g., initial and follow-up TB prevalence surveys), effect on health systems, and cost-effectiveness (including averted long-term TB treatment costs) as key outcomes.

As with any model-based analysis, our study has certain limitations. Our model structure makes a number of simplifying assumptions (e.g., homogeneous mixing, constant rate of case detection) for purposes of conveying a transparent message with a minimum of parameter inputs. Although our results were reasonably robust to variations in parameter values, our sensitivity analyses cannot guarantee that our model’s structure reflects the dynamics of TB in Karachi. Furthermore, this model evaluates a complex, multifaceted campaign centered at a new hospital, and assumes that the effect of such an intervention could be sustained over a five-year period (either through residual impact or ongoing efforts). We similarly assume that the proportion of false-positive notifications (e.g., empiric treatments without bacteriological confirmation) did not change during the intervention; if all additional smear-negative, smear-uncertain, and extrapulmonary notifications were false positive, our estimates overestimate true impact by a factor of two. Since we modeled the intervention as a comprehensive package of activities, we are unable to isolate the individual impact of specific components, and our projections will overestimate the impact of interventions that cannot be sustained over time. Since our model relies on notification data, our accounting of informal-sector activities is incomplete, although assumptions about the size of the informal sector did not drive our conclusions. We did not explicitly consider the possibility that some additional notifications may have arisen from individuals who migrated from outside the study area, though the intervention focused its communications and case-finding efforts within the study area only. We also did not explicitly model drug-resistant TB; if case-finding strategies are implemented without drug susceptibility testing, the proportion of drug-resistant TB may increase over time, with substantial economic and epidemiological consequences. Our age structure was simplistic and thus overestimated the number of individuals who matured into the adult class by 1.5% by the end of 2015; since these individuals have lower burden of latent infection and are thus more likely to benefit from the intervention, we may have slightly underestimated the impact of the intervention by adopting this simplifying assumption. Finally, approaches to improved TB case-finding must to be tailored to local conditions; the impact of this unique intervention may not fully generalize to other epidemiological or sociodemographic settings. Particularly, in areas where existing passive case-finding is stronger than in Karachi, the impact of additional case-detection activities may not be as profound as seen in this analysis.

In conclusion, this dynamic transmission model suggests that a multifaceted and sustained TB case-finding intervention in Karachi, Pakistan, could reasonably reduce TB incidence and mortality, respectively, by 25% and 50% cumulatively over five years, or by 45% and >70% at the end of a five-year period. Even if the case-finding intervention could not be sustained beyond one year or alternatively, if most non-confirmed cases were false positive, half of the cumulative five-year impact on incidence and mortality could still be attained. Though we could not isolate the effect of any single component of this intervention, these projections were relatively robust to variations in parameter estimates, including assumptions about the size of the informal sector. Our results underscore the urgent importance of deploying and evaluating novel approaches to TB case-finding in Asian megacities and other areas of concentrated TB transmission. In these areas, strategic investments in innovative, intensive, and sustained case-finding initiatives may reasonably be expected to achieve marked results within five years, making local elimination of TB an attainable goal. 

## Supporting Information

Supporting Information S1 **Figure S1: Relationship of Parameters to Transmission Model of TB Epidemic in Karachi.** This figure provides a visual perspective on the parameters used in the model and is not designed to be a mathematically complete formulation (as appears in the following sections). Each set of parameters must be multiplied by the size of the preceding box to obtain the corresponding rate of change. Mortality from TB (μ_sp_ and μ_sn_) is included in the model but not shown here. **Table S1**. **Model Parameters**. Shown are all model parameters used, with corresponding values and references. **Figure S2: Distribution of ages (in years) within the TB model**. The age distribution was derived through the convolution of all 9 exponentially distributed age classes, the first having a mean of 10 years and the subsequent 8 with a mean of 5 years. **Table S2: Fit for high and low incidence scenarios, 2008-2011**. Shown are the fitted parameter values for high and low incidence scenarios, respectively. **Table S3: Duration of active TB (years) in each model year including self-cure and mortality**. These data correspond to the prevalence/incidence ratio for each form of active TB, in each year shown. **Table S4: Ranges of rate of detection and treatment used in sensitivity analyses**.
(PDF)
