# Supplementary material for: Population-Level Impact of Active Tuberculosis Case Finding in an Asian Megacity
Source: PLoS One. 2013 Oct 16;8(10):e77517. doi: 10.1371/journal.pone.0077517 (PMC3797738; doi:10.1371/journal.pone.0077517)
Supplement: Supporting Information S1 — Figure S1: Relationship of Parameters to Transmission Model of TB Epidemic in Karachi. This figure provides a visual perspective on the parameters used in the model and is not designed to be a mathematically complete formulation (as appears in the following sections). Each set of parameters must be multiplied by the size of the preceding box to obtain the corresponding rate of change. Mortality from TB (μsp and μsn) is included in the model but not shown here. Table S1. Model Parameters. Shown are all model parameters used, with corresponding values and references. Figure S2: Distribution of ages (in years) within the TB model. The age distribution was derived through the convolution of all 9 exponentially distributed age classes, the first having a mean of 10 years and the subsequent 8 with a mean of 5 years. Table S2: Fit for high and low incidence scenarios, 2008-2011. Shown are the fitted parameter values for high and low incidence scenarios, respectively. Table S3: Duration of active TB (years) in each model year including self-cure and mortality. These data correspond to the prevalence/incidence ratio for each form of active TB, in each year shown. Table S4: Ranges of rate of detection and treatment used in sensitivity analyses. (PDF) [file pone.0077517.s001.pdf]

Population-Level Impact of Active Tuberculosis Case Finding in an Asian Megacity
Supplemental Text

David W. Dowdy, Ismat Lotia, Andrew S. Azman,
Jacob Creswell, Suvanand Sahu, Aamir Khan

Section S1 Model Description

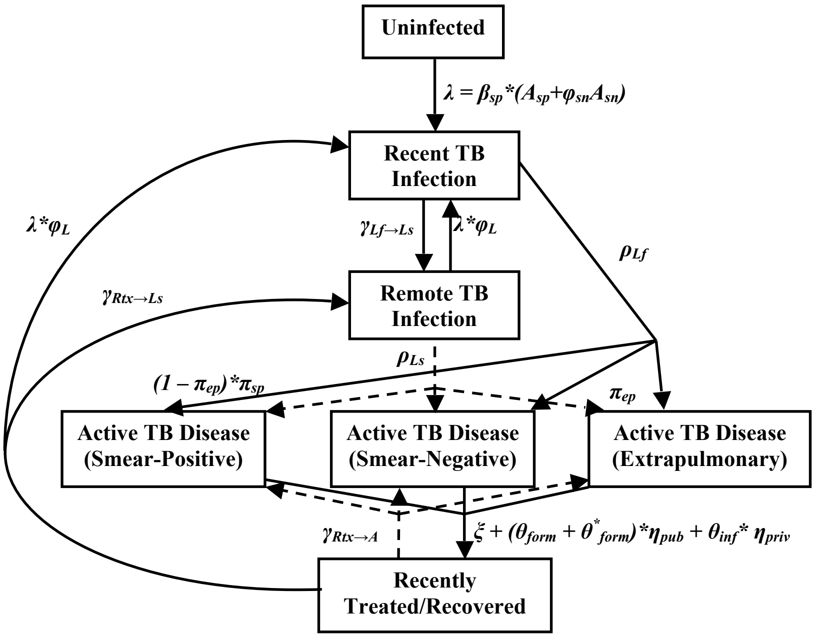

Figure S1: **Relationship of Parameters to Transmission Model of TB Epidemic in Karachi.** This figure provides a visual perspective on the parameters used in the model and is not designed to be a mathematically complete formulation (as appears in the following sections). Each set of parameters must be multiplied by the size of the preceding box to obtain the corresponding rate of change. Mortality from TB (μ_{sp} and μ_{sn}) is included in the model but not shown here.

Table S1: **Model Parameters**

**Parameter**	**Value**	**Description**	**Source**
β_{sp}	fit	Smear positive (SP) transmission parameter	[10]
ϕ_{sn}	0.22	Relative infectiousness of smear negative (SN) TB	[2]
ϕ_L	0.5	Protection of latent TB against reinfection	[9, 1, 7]
π_{sp}	0.65	Proportion of pulmonary TB that is SP	study data, [5, 6]
π_{ep}	0.24	Proportion of TB that is extrapulmonary (EP)	study data
μ_{sp}	0.233	Mortality rate from SP TB, yearly	[8]
μ_{sn}	$0.286 * 0.233$	Mortality rate from SN and EP TB, yearly	[8]
ζ_{sp}	0.1	Self-cure rate from SP, yearly	[8]
ζ_{sn}	$2.7 * 0.1$	Spontaneous-cure rate from SN and EP, yearly	[8]
ρ_{Lf}	0.07	Rate of (rapid) progression after recent infection, yearly	[9]
ρ_{Ls}	0.0005	Rate of (slow) reactivation after remote infection, yearly	[3]
ρ_{rel}	fit	Rate of relapse following recent treatment, yearly	study data
$\gamma_{Lf \to Ls}$	0.5	Rate of progression (stabilization) from recent infection to remote infection, yearly	[9]
$\gamma_{Rtx \to Ls}$	0.2	Rate of progression (stabilization) from recent treatment to remote infection, yearly	[9]
$\gamma_{Rtx \to Asp}$	$\rho_{rel}\pi_{sp}(1-\pi_{ep})$	Rate of reinfection followed by progression to active TB for recently treated to SP TB	derived from other parameters
$\gamma_{Rtx \to Asn}$	$\rho_{rel}(1-\pi_{sp})(1-\pi_{ep})$	Rate of reinfection followed by progression for recently treated to SN TB	derived from other parameters
$\gamma_{Rtx \to Aep}$	$\rho_{rel}\pi_{ep}$	Rate of reinfection followed by progression to active TB for recently treated to EP TB	derived from other parameters
$\theta_{form,sp}$	fit	Annual rate of formal sector SP cases notified	study data
$\theta_{form,sn}$	fit	Annual rate of formal sector SN cases notified	study data
$\theta_{form,ep}$	fit	Annual rate of formal sector EP cases notified	study data
$\theta^{\star}_{form,\bullet}$	fit	Annual increase in rate of formal sector ($\bullet$) TB after intervention	study data
$\theta_{inf,\bullet}$	$0.455(\theta_{form,\bullet})$	Annual rate of ($\bullet$) TB diagnosis in informal sector 2008-2010	study data
η_{form}	$0.815^{\dagger}$	Formal sector treatment success probability	study data
η_{inf}	0.5	Informal sector treatment success probability	[4]
δ_1	1/10	Mean time spent in age category 1, (0-9 y/o)	assumed
δ_i	1/5	Mean time spent in all other age classes (ages 10 to 49)	assumed
ν	0.03	Annual population growth rate	assumed

† Calculated by $\frac{0.75}{0.92}$; the treatment success probability (0.75) divided by the probability of not dying while on treatment (0.92).

Section S1.1 Model Equations

This is the system for a generic age group, i[1]:

$$\lambda = \beta_{sp}(\sum_{i=2}^{9} A_{sp,i} + \phi_{sn} A_{sn,i}) \tag{1}$$

$$\begin{aligned}\frac{dS_i}{dt} =& S_i\left(\nu - \lambda - \delta_i\right) + \mathbb{I}_{i\neq 1}\delta_{i-1}S_{i-1} + \\ & \mathbb{I}_{i=1}\big(\delta_9(S_9 + Lf_9 + Ls_9 + Asp_9 + Asn_9 + Aep_9 + Rtx_9) + \\ & \sum_i(\mu_{sp}Asp_i + \mu_{sn}Asn_i + \mu_{sn}Aep_i)\big)\end{aligned} \tag{2}$$

$$\frac{dLf_i}{dt} = S_i\lambda + \lambda\phi_L(Ls_i + Rtx_i) + Lf_i(\nu - \mathbb{I}_{i\neq 1}\rho_{Lf} - \gamma_{Lf\to Ls} - \delta_i) + \mathbb{I}_{i\neq 1}\delta_{i-1}Lf_{i-1} \tag{3}$$

$$\frac{dLs_i}{dt} = Lf_i\gamma_{Lf\to Ls} + +Rtx_i\gamma_{Rtx\to Ls} + Ls_i(\nu - \mathbb{I}_{i\neq 1}\rho_{Ls} - \delta_i - \lambda\phi_L) + \mathbb{I}_{i\neq 1}\delta_{i-1}Ls_{i-1} \tag{4}$$

$$\begin{aligned}\frac{dAsp_i}{dt} =& \mathbb{I}_{i\neq 1}\big(\pi_{sp}(1-\pi_{ep})\left(Lf_i\rho_{Lf} + Ls_i\rho_{Ls}\right) + Rtx_i\rho_{rel}\pi_{sp}(1-\pi_{ep}) + \delta_{i-1}Asp_{i-1}\big) + \\ & Asp_i\left(\nu - \mu_{sp} - \delta_i - (\theta_{form,sp} + \theta^{\star}_{form,sp})\eta_{form} - \theta_{inf,sp}\eta_{inf} - \zeta_{sp}\right)\end{aligned} \tag{5}$$

$$\begin{aligned}\frac{dAsn_i}{dt} =& \mathbb{I}_{i\neq 1}\big((1-\pi_{sp})(1-\pi_{ep})\left(Lf_i\rho_{Lf} + Ls_i\rho_{Ls}\right) + Rtx_i\rho_{rel}(1-\pi_{sp})(1-\pi_{ep}) + \delta_{i-1}Asn_{i-1}\big) + \\ & Asn_i\left(\nu - \mu_{sn} - \delta_i - (\theta_{form,sn} + \theta^{\star}_{form,sn})\eta_{form} - \theta_{inf,sn}\eta_{inf} - \zeta_{sn}\right)\end{aligned} \tag{6}$$

$$\begin{aligned}\frac{dAep_i}{dt} =& \mathbb{I}_{i\neq 1}\big(\pi_{ep}\left(Lf_i\rho_{Lf} + Ls_i\rho_{Ls}\right) + Rtx_i\rho_{rel}\pi_{ep} + \delta_{i-1}Aep_{i-1}\big) + \\ & Aep_i\left(\nu - \mu_{sn} - \delta_i - (\theta_{form,ep} + \theta^{\star}_{form,ep})\eta_{form} - \theta_{inf,ep}\eta_{inf} - \zeta_{sn}\right)\end{aligned} \tag{7}$$

$$\begin{aligned}\frac{dRtx_i}{dt} =& Aep_i\left((\theta_{form,ep} + \theta^{\star}_{form,ep})\eta_{form} + \theta_{inf,ep}\eta_{inf} + \zeta_{sn}\right) + \\ & Asn_i\left((\theta_{form,sn} + \theta^{\star}_{form,sn})\eta_{form} + \theta_{inf,sn}\eta_{inf} + \zeta_{sn}\right) + \\ & Asp_i\left((\theta_{form,sp} + \theta^{\star}_{form,sp})\eta_{form} + \theta_{inf,sp}\eta_{inf} + \zeta_{sp}\right) + \\ & Rtx_i(\nu - \delta_i - \rho_{rel} - \gamma_{Rtx\to Ls} - \lambda\phi_L) + \mathbb{I}_{i\neq 1}\delta_{i-1}Rtx_{i-1}\end{aligned} \tag{8}$$

Section S1.2 Model Text

As shown in Figure S1, the model divides the population into seven compartments based on TB status: uninfected (S_i), recently infected (Lf_i), remotely infected (Ls_i), active smear-positive pulmonary (Asp_i), active smear-negative pulmonary (Asn_i), active extrapulmonary (Aep_i), and recently treated (Rtx_i).

These compartments are further sub-divided into nine age strata, the first (age 0-9) with an exit rate of $\frac{1}{10}$ year^{-1} (i.e., mean duration of 10 years), and the following eight each with an exit rate of $\frac{1}{5}$ year^{-1}. Thus, the lifespan of individuals in the model has a mean of 50 years and a standard deviation of 17.3 (see age distribution in Figure S2).

[1] where $\mathbb{I}_{i\neq 1}$ is an indicator function equal to 1 when the condition $i \neq 1$ is true

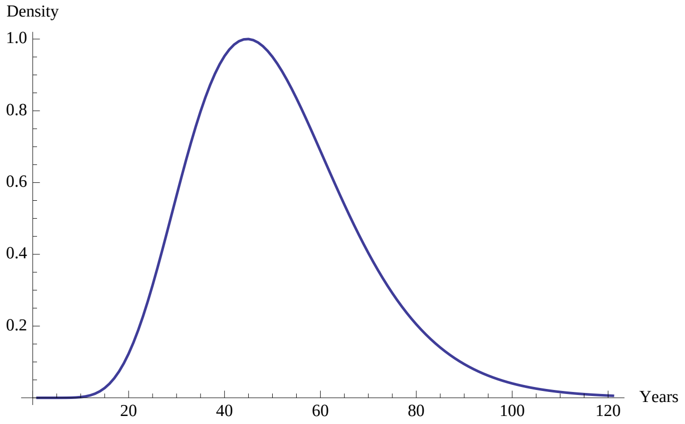

Figure S2: Distribution of ages (in years) within the TB model. The age distribution was derived through the convolution of all 9 exponentially distributed age classes, the first having a mean of 10 years and the subsequent 8 with a mean of 5 years.

Model equations are given above. This section provides a narrative description and justification of each equation. Where parameter values are given, these represent the values for the main model; all parameter values were varied widely in sensitivity analysis.

Equation 1: Force of Infection, λ
The force of infection (λ) is defined as the product of the annual transmission rate (β_{sp}) and the sizes of the active pulmonary TB compartments (Asp_i and Asn_i). Smear-negative pulmonary TB is assumed to be infectious, though at a reduced level of ϕ_{sn} (22%) relative to smear-positive pulmonary TB. Fully extrapulmonary TB (Aep_i) is assumed to be non-infectious, as is pediatric TB ($i = 1$). Patients with TB that is both pulmonary and extrapulmonary are infectious and therefore classified according to their pulmonary manifestations.

Equation 2: Uninfected, S_i
Uninfected individuals can be infected with TB (λ), or age to the next-oldest stratum (δ_i). Additions to the uninfected compartment include population growth (ν) and aging from the next-youngest stratum (δ_{i-1}). Furthermore, to maintain a stable population in the absence of any population growth, it is also necessary that births equal deaths; births occur entirely into the uninfected compartment, age stratum 1 (i.e., age 0-9). Thus, the compartment of uninfected individuals in age stratum 1, also grows by an amount equal to the number of deaths due to aging (δ_9*[all compartments with $i = 9$]) as well as TB mortality ($\sum_i[\mu_{sp}Asp_i + \mu_{sn}Asn_i + \mu_{sn}Aep_i]$). Note that population growth, ν, which can occur by such means as immigration, is assumed to occur across all compartments equally, but that deaths - which are far more common - are replaced entirely by births into the uninfected compartment, age stratum 1.

Equation 3: Recently Infected, Lf_i
All individuals who are infected with TB including uninfected individuals ($S_i\lambda$), as well as remotely-infected and recently-treated individuals ($\lambda\phi_L[Ls_i + Rtx_i]$), transfer into this compartment, which lasts 2 years following infection. Individuals who have been previously infected with TB are presumed to have partial immunological protection ($\phi_L = 50\%$) against the process of reinfection (i.e., transfer to recently infected), whether by reduction in infection risk or reduction in risk of progression to active TB. As above, population growth (ν) occurs across all compartments.

Individuals leave this compartment through rapid progression to active TB ($\rho_{Lf}Lf_i$), which occurs at a much higher rate (7%/year) than does reactivation from remote infection (0.05%/year), reflecting the increased risk of TB progression within the 2 years following infection. Since this is a model of adult TB, we do not allow primary progression to active TB from the 0-9 year age stratum (i.e., $\mathbb{I}_{i\neq 1}$), but we do track children through the recently infected compartment; thus, for example, a child infected at age 9 would still experience

increased risk of developing adult-form TB on reaching his/her 10th birthday. This also allows for a realistic distribution of latent infection among individuals entering the period at risk for adult TB upon reaching age 10.

In competition to the rate of progression to active TB is the rate of stabilization ($\gamma_{Lf \to Ls}$) to remote infection, which occurs at a rate of 0.5 year^{-1} (i.e., mean 2-year compartment duration), after which the rate of progression to active TB drops substantially. Ultimately, $0.07/(0.5 + 0.07) = 12\%$ of individuals will progress rapidly to active TB rather than stabilize. Individuals also transfer out (but to another recently-infected compartment) through aging (δ_i) to the next-oldest age stratum and transfer in through aging (δ_{i-1}) from the next-youngest age stratum.

Equation 4: Remotely Infected, Ls_i
Individuals transfer into this compartment primarily by stabilization of recent infection ($\gamma_{Lf \to Ls}$) or recent treatment ($\gamma_{Rtx \to Ls}$). Prior to stabilization, the recent infection compartment experiences an increased risk of rapid progression to active TB (for 2 years), and the recent treatment compartment experiences an increased risk of relapse (for 5 years, to fully account for any paradoxical increases in relapse rates after increasing the rate of case-finding and treatment over the 5-year analysis period). After stabilization, the rate of reactivation to active TB (ρ_{Ls}) is small (0.05%/year), and is not allowed for the first age stratum (i.e., $\mathbb{I}_{i \neq 1}$). In addition to stabilization and reactivation, people transfer into this compartment through population growth (ν), out through aging to the next-oldest age stratum (δ_i), out through reinfection to recent infection ($\lambda \phi_L$), and in through aging from the next-youngest age stratum (δ_{i-1}).

Equations 5-7: Active TB, smear-positive pulmonary (Asp_i), smear-negative pulmonary (Asn_i), and extrapulmonary (Aep_i)
Individuals enter these compartments through rapid progression from recent infection ($Lf_i \rho_{Lf}$), reactivation from remote infection ($Ls_i \rho_{Ls}$), and relapse from recent treatment ($Rtx_i \rho_{rel}$); the three equations differ in the proportion of these individuals who enter each compartment of active TB. Specifically, the proportion entering the extrapulmonary compartment (equation 7) is π_{ep}, the proportion entering the smear-positive pulmonary compartment (equation 5) is $\pi_{sp}(1 - \pi_{ep})$, and the proportion entering the smear-negative pulmonary compartment (equation 6) is $(1 - \pi_{sp})(1 - \pi_{ep})$. These processes are not incorporated for children in the first age stratum, as above.

The primary mode of transition out of these compartments is through diagnosis and treatment, the rate of which for each form of TB (smear-positive, smear-negative, and extrapulmonary) is fit to specific data from the study site. Thus, for smear-positive pulmonary TB (equation 5), individuals are detected at rate of $\theta_{form,sp}$ in the formal sector and $\theta_{inf,sp}$ in the informal sector. The corresponding rates of detection for smear-negative pulmonary TB (equation 6) are $\theta_{form,sn}$ and $\theta_{inf,sp}$; and for extrapulmonary TB (equation 7) are $\theta_{form,ep}$ and $\theta_{inf,sp}$. TB treatment may be unsuccessful due to default, failure, or transfer out of the system; these events are modeled as individuals remaining in the active TB compartment. Thus, the rate of exit due to case detection θ is multiplied by a treatment success term η, which differs according to treatment in the formal or informal sector (η_{form} and η_{inf}, respectively), but not by clinical manifestation. Since only those individuals who were started on therapy were notified, 'initial default' is implicitly modeled as a reduction in the diagnostic rate rather than being explicitly modeled in the treatment successes proportion. Notably, deaths on treatment are traditionally considered treatment failures, but for purposes of this model these are considered late diagnoses, meaning that we assume death to occur before diagnosis in these individuals. As such, they are incorporated into the TB mortality rate rather than the treatment success proportion.

TB mortality is explicitly incorporated as a rate of exit from each compartment: μ_{sp} for smear-positive pulmonary TB, and μ_{sn} for smear-negative TB (whether pulmonary or extrapulmonary). Similarly, individuals who do not receive treatment may spontaneously resolve their TB disease; this is modeled as a rate ζ_{sp} for smear-positive TB and ζ_{sn} for smear-negative TB.

In addition to the processes above, people transfer into the active TB compartments through population growth (ν), out through aging to the next-oldest age stratum (δ_i), and in through aging from the next-youngest age stratum (δ_{i-1}).

Equation 8: Recently Treated, Rtx_i
In the first years after successful treatment (or spontaneous resolution), individuals remain at high risk for relapse to active TB. Thus, active case-finding interventions in a given year can have a paradoxical effect on TB incidence in future years, as more individuals are treated and therefore pose a risk of developing recurrent TB. Therefore, individuals with active TB who are successfully treated (or spontaneously resolve) do not progress to remote infection, but rather to a recently treated compartment. Individuals enter this compartment through successful treatment in the formal sector ($\theta_{form}\eta_{form}$), successful treatment in the informal sector ($\eta_{\inf}\theta_{inf}$), or spontaneous recovery (ζ); each of these rates is dependent on the corresponding form of TB (smear-positive, smear-negative pulmonary, or extrapulmonary). Individuals leave the recently-treated compartment through relapse to active TB (ρ_{rel}) or through reinfection ($\lambda\phi_L$). Individuals may also stabilize to the remote-infection compartment ($\gamma_{Rtx\rightarrow Ls}$); this process occurs at a rate of 0.2 year^{-1} (i.e., mean 5-year compartment duration), such that anyone who is detected through the intervention will remain at increased risk of relapse throughout the analysis period (a conservative assumption). In addition to the processes above, people transfer into the recent treatment compartment through population growth (ν), out through aging to the next-oldest age stratum (δ_i), and in through aging from the next-youngest age stratum (δ_{i-1}).

Section S2 Model Fitting

We fit the model to data from the intervention in Karachi as follows. First, based on data from the World Health Organization, we assumed that the TB epidemic in Pakistan was at steady-state in 2008, and that the local situation in the intervention area reflected this steady-state assumption. We then used two separate procedures for model fitting that gave very similar results. The first procedure (and the one reflected in the main manuscript text) was an iterative routine that matched one model parameter to one data element in one-to-one fashion. Thus, the number of transmissions arising from a smear-positive case each year (β_{sp}) was matched to TB incidence, the relapse rate (ρ_{rel}) was matched to the proportion of cases identified as retreatment cases, and the formal-sector diagnostic rates ($\theta_{form,sp}$; $\theta_{form,sn}$; and $\theta_{form,ep}$) were matched to the type-specific notification rates in the intervention area in Karachi. The model parameters were iteratively fit until the model matched each data element to all significant figures, and equilibrium (defined as no more than 1% or 1 person change in any compartment size over a 5-year period) was achieved. The second procedure minimized an objective function on the basis of the same five model parameters; this function was defined as the sum of $\frac{(observed-expected)^2}{expected}$ for the five model parameters. Table S2 shows the values of the parameters obtained through the same (former) procedure as the main text.

Since notification rates in the intervention rose from 2008 through 2010 before the intervention was initiated, we did not assume equilibrium during that time. Rather, once the equilibrium model was defined in 2008 as above, we fit a linear increase in the formal-sector diagnostic rates to the observed increases in type-specific case notifications in the years 2009 and 2010. Because of the weather-based seasonality of TB notifications in Karachi, we did not seek to fit month-over-month notification rates. The rate of increase in formal-sector diagnostic rates was defined to minimize:

$$\left|\text{modeled '09 notifications} - \text{observed '09 notifications}\right| + \left|\text{modeled '10 notifications} - \text{observed '10 notifications}\right|,$$

for each type of TB (smear-positive, smear-negative, and extrapulmonary). During this time, we assume that informal-sector diagnoses of TB remained constant.

Starting on January 1, 2011, we modeled the intervention as a linear increase in the type-specific diagnostic rates, and fit this to the observed number of formal-sector TB case notifications in that year. Adopting a maximally conservative position (from the point of intervention impact), we assumed a simultaneous linear decrease in the informal-sector TB diagnostic rate, reaching zero on January 1, 2012. Thus, in the intervention scenario, by the end of 2011, TB case notifications through the formal sector were assumed to include cross-over of all individuals who otherwise would have been diagnosed in the informal sector. To the extent that individuals were still diagnosed in the informal sector, the overall incidence of TB would be

lower, and the modeled impact of the intervention larger. From 2012 through 2016, we assumed that the TB diagnostic rate remained stable at the level achieved by January 1, 2012. Although further increases in TB diagnostic rate are likely (should the intervention be continued at equivalent intensity), we did not model any additional increases in diagnostic rate beyond 2011, for purposes of creating a clean comparison between intervention and counterfactual scenarios.

To construct the counterfactual scenario, we assumed that informal-sector TB diagnosis remained at the same rate as at the end of 2010. Since it is unlikely that formal-sector TB diagnosis would immediately stabilize, and yet we wanted to provide a fair comparison against the intervention (i.e., stable diagnostic rates in 2012 and beyond), we gradually increased the TB diagnostic rate in 2011 as a function of time t from January 1, 2011. Thus, the slope of increase in the diagnostic rate at any given time t during 2011, given the slope of increase $\theta^\star$ in the diagnostic rate in 2009-2010, was defined as $\theta^\star(2012 - t)$, such that the slope of increase was $\theta^\star$ on January 1, 2011, and zero (i.e., stable) on January 1, 2012.

Table S2: Fit for high and low incidence scenarios, 2008-2011.

incidence	year	β_{sp}	ρ_{rel}	$\theta_{form,sp}$	$\theta^\star_{form,sp}$	$\theta_{form,sn}$	$\theta^\star_{form,sn}$	$\theta_{form,ep}$	$\theta^\star_{form,ep}$
231	2008	16.1	0.024	0.41	0	0.16	0	0.50	0
231	2009				0.22		0.074		0.29
231	2010				0.22		0.074		0.29
231	2011				1.94		1.26		2.04
350	2008	13.8	0.029	0.19	0	0.090	0	0.22	0
350	2009				0.09		0.040		0.11
350	2010				0.09		0.040		0.11
350	2011				0.53		0.58		0.46

Note: $\theta^\star$'s represent the value at the end of the year with the rate linearly fit from the previous year's rate.

Table S3: Duration of active TB (years) in each model year including self-cure and mortality.

incidence	year	Smear Positive	Smear Negative	Extra Pulmonary
231	2008	1.4	2.0	2.0
231	2009	1.0	1.8	1.3
231	2010	1.0	1.8	1.3
231	2011	0.4	0.6	0.4
350	2008	1.9	2.3	4.5
350	2009	1.6	2.1	3.0
350	2010	1.6	2.1	3.0
350	2011	1.0	1.0	1.5

Section S3 Uncertainty Analyses

Table S4: Ranges of rate of detection and treatment used in sensitivity analyses.

**parameter**	**range**
primary analysis, 2009-2010	
$\theta^{\star}_{form,sp}$	0.16-0.28
$\theta^{\star}_{form,sn}$	0.05-0.1
$\theta^{\star}_{form,ep}$	0.23-0.35
primary analysis, 2011	
$\theta^{\star}_{form,sp}$	1.4-2.5
$\theta^{\star}_{form,sn}$	0.8-1.8
$\theta^{\star}_{form,ep}$	1.5-2.5
high-incidence analysis, 2009-2010	
$\theta^{\star}_{form,sp}$	0.06-0.12
$\theta^{\star}_{form,sn}$	0.025-0.055
$\theta^{\star}_{form,ep}$	0.08-0.14
high-incidence analysis, 2011	
$\theta^{\star}_{form,sp}$	0.35-0.75
$\theta^{\star}_{form,sn}$	0.4-0.8
$\theta^{\star}_{form,ep}$	0.3-0.6

References

[1] Andrews, J. R., Noubary, F., Walensky, R. P., Cerda, R., Losina, E., and Horsburgh, C. R. Risk of progression to active tuberculosis following reinfection with Mycobacterium tuberculosis. *Clinical Infectious Diseases 54*, 6 (Mar. 2012), 784–791.

[2] Behr, M. A., Warren, S. A., Salamon, H., Hopewell, P. C., Ponce de Leon, A., Daley, C. L., and Small, P. M. Transmission of Mycobacterium tuberculosis from patients smear-negative for acid-fast bacilli. *The Lancet 353*, 9151 (Feb. 1999), 444–449.

[3] Horsburgh, C. R., O'Donnell, M., Chamblee, S., Moreland, J. L., Johnson, J., Marsh, B. J., Narita, M., Johnson, L. S., and von Reyn, C. F. Revisiting rates of reactivation tuberculosis: a population-based approach. *American journal of respiratory and critical care medicine 182*, 3 (Aug. 2010), 420–425.

[4] Lönnroth, K., Thuong, L. M., Lambregts, K., Quy, H. T., and Diwan, V. K. Private tuberculosis care provision associated with poor treatment outcome: comparative study of a semi-private lung clinic and the NTP in two urban districts in Ho Chi Minh City, Vietnam. National Tuberculosis Programme. *Int J Tuberc Lung Dis 7*, 2 (Feb. 2003), 165–171.

[5] Steingart, K. R., Henry, M., Ng, V., Hopewell, P. C., Ramsay, A., Cunningham, J., Urbanczik, R., Perkins, M., Aziz, M. A., and Pai, M. Fluorescence versus conventional sputum smear microscopy for tuberculosis: a systematic review. *The Lancet Infectious Diseases 6*, 9 (Sept. 2006), 570–581.

[6] Steingart, K. R., Ng, V., Henry, M., Hopewell, P. C., Ramsay, A., Cunningham, J., Urbanczik, R., Perkins, M. D., Aziz, M. A., and Pai, M. Sputum processing methods to improve the sensitivity of smear microscopy for tuberculosis: a systematic review. *The Lancet Infectious Diseases 6*, 10 (Oct. 2006), 664–674.

[7] Sutherland, I., Svandová, E., and Radhakrishna, S. The development of clinical tuberculosis following infection with tubercle bacilli. 1. A theoretical model for the development of clinical tuberculosis following infection, linking from data on the risk of tuberculous infection and the incidence of clinical tuberculosis in the Netherlands. *Tubercle 63*, 4 (Dec. 1982), 255–268.

[8] Tiemersma, E. W., van der Werf, M. J., Borgdorff, M. W., Williams, B. G., and Nagelkerke, N. J. D. Natural History of Tuberculosis: Duration and Fatality of Untreated Pulmonary Tuberculosis in HIV Negative Patients: A Systematic Review. *PLoS ONE 6*, 4 (2011), e17601.

[9] Vynnycky, E., and Fine, P. E. The natural history of tuberculosis: the implications of age-dependent risks of disease and the role of reinfection. *Epidemiology and Infection 119*, 2 (Oct. 1997), 183–201.

[10] World Health Organization. WHO Report 2011: Global Tuberculosis Control. 1–258.
